# Prebiotic effects of yeast mannan, which selectively promotes *Bacteroides thetaiotaomicron* and *Bacteroides ovatus* in a human colonic microbiota model

**DOI:** 10.1038/s41598-020-74379-0

**Published:** 2020-10-15

**Authors:** Shunsuke Oba, Tadahiro Sunagawa, Reiko Tanihiro, Kyoko Awashima, Hiroshi Sugiyama, Tetsuji Odani, Yasunori Nakamura, Akihiko Kondo, Daisuke Sasaki, Kengo Sasaki

**Affiliations:** 1 Core Technology Laboratories, Asahi Quality & Innovations, Ltd., 1-21, Midori 1-Chome, Moriya-Shi, 302-0106 Japan; 2 Graduate School of Science, Technology and Innovation, Kobe University, 1-1 Rokkodai-cho, Nada-ku, Kobe, Hyogo 657-8501 Japan

**Keywords:** Carbohydrates, Microbial ecology, Applied microbiology, Microbial communities, Dietary carbohydrates, Polysaccharides

## Abstract

Yeast mannan (YM) is an indigestible water-soluble polysaccharide of the yeast cell wall, with a notable prebiotic effect on the intestinal microbiota. We previously reported that YM increased *Bacteroides thetaiotaomicron* abundance in in vitro rat faeces fermentation, concluding that its effects on human colonic microbiota should be investigated. In this study, we show the effects of YM on human colonic microbiota and its metabolites using an in vitro human faeces fermentation system. Bacterial 16S rRNA gene sequence analysis showed that YM administration did not change the microbial diversity or composition. Quantitative real-time PCR analysis revealed that YM administration significantly increased the relative abundance of *Bacteroides ovatus* and *B. thetaiotaomicron*. Moreover, a positive correlation was observed between the relative ratio (with or without YM administration) of *B. thetaiotaomicron* and *B. ovatus* (r = 0.92), suggesting that these bacteria utilise YM in a coordinated manner. In addition, YM administration increased the production of acetate, propionate, and total short-chain fatty acids. These results demonstrate the potential of YM as a novel prebiotic that selectively increases *B. thetaiotaomicron* and *B. ovatus* and improves the intestinal environment. The findings also provide insights that might be useful for the development of novel functional foods.

## Introduction

**Figure 1:**
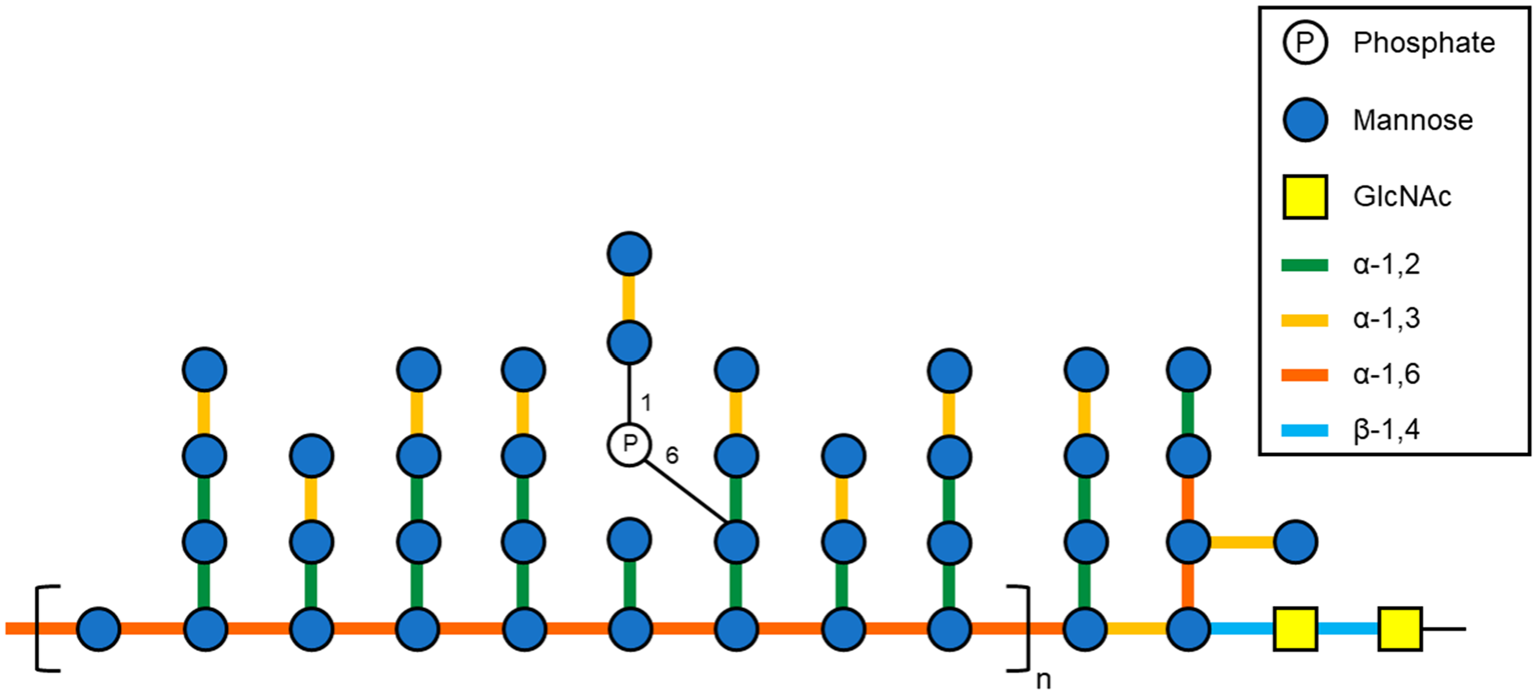
Schematic of the yeast mannan (YM) structure. YM includes a linear α-1, 6-mannoside backbone branched with α-1, 2-mannoside and α-1, 3-mannoside bonds in the form of mono-, di-, tri-, and tetramers.

Yeast has been widely consumed since ancient times in fermented foods and beverages such as bread, beer, and wine. Yeast mannan (YM) is an indigestible water-soluble polysaccharide of the yeast cell wall that has rarely been used as a food ingredient. YM is a densely branched α-linked mannose polymer with a molecular weight ranging from 20,000 to 200,000 Da^1^. It includes a linear α-1, 6-mannoside backbone branched with α-1, 2-mannoside and α-1, 3-mannoside bonds in the form of mono-, di-, tri-, and tetramers (Fig. 1)^1,2^. This structure is distinct from other plant cell wall mannans, such as konjac glucomannan and carob galactomannan, which include only β-linked mannose and not α-linked mannose^3^. YM has various effects in cells and mouse models, including immunomodulation, wound repair, and anti-inflammatory effects, and has potential health benefits in humans and animals^4–6^. YM appears to be utilised by specific intestinal bacteria due to its elaborate structure^7^, and the impacts of YM on the intestinal microbiota ecosystem have attracted research attention.

The human intestinal tract is colonised by trillions of microorganisms, which greatly contribute to host health by providing nutrients, energy, pathogen resistance, and immune response modulation^8–11^. Due to its importance in homeostasis, dysbiosis of the intestinal microbiota is associated with various multifactorial diseases, including metabolic, inflammatory bowel, cardiovascular, neoplastic, and neurological diseases^11–15^. Therefore, controlling the composition of the intestinal microbiota and maintaining a favourable intestinal environment with the diet plays a key role in maintaining the host’s health. Bacteroidetes, which is composed largely of members of the genus *Bacteroides*, is a dominant gut-associated bacterial phylum in healthy adult microbiota^16^ using a glycan-acquisition strategy; Bacteroidetes members employ multiple cell envelope-associated protein complexes called the starch utilisation system (Sus)^17^. Sus is the most studied glycan degradation system encoded by a polysaccharide utilisation locus (PUL)^18^. The proteins in the Sus are located in the outer membrane and periplasm of the cell. On the cell surface, the proteins bind glycan and degrade it into oligosaccharides and transport these oligosaccharides into the periplasm, where they are broken down into smaller saccharides and imported into the cell^7,18–20^. By utilising Sus-like systems, Bacteroidetes can degrade various indigestible carbohydrates from the human diet^21–23^. This process leads to the production of short-chain fatty acids (SCFAs)^24^, which act as both nutrients and energy sources for the host^25^.

*Bacteroides thetaiotaomicron*, a prominent human intestinal symbiont in the phylum Bacteroidetes, exhibits various distinctive functions, including anti-rotavirus activity^26^, induction of matrilysin^27^, and attenuation of colon inflammation^28^. Furthermore, clinical trials have been conducted using *B. thetaiotaomicron* as a live biotherapeutic candidate for the treatment of Crohn’s disease^29^. *B. thetaiotaomicron* is believed to confer various benefits on host health; therefore, it is desirable to increase its abundance. *B. thetaiotaomicron* has various PULs, including genomic *sus* genes, and thereby a broad ability to digest dietary fibre polysaccharides and host glycans^30^. YM is a polysaccharide that is digested and utilised by *B. thetaiotaomicron*, which metabolises it through a ‘selfish mechanism’ due its unique property of having three mannan-specific PULs; most other bacteria do not have such PULs and therefore cannot utilise it^7^. Unlike traditional prebiotics such as inulin, fructo-oligosaccharides, and galacto-oligosaccharides which increase *Lactobacillus* and *Bifidobacterium*^31^, we consider YM to be a valuable ingredient as a novel prebiotic candidate that increases the abundances of *B. thetaiotaomicron* and other *Bacteroides* spp. YM utilisation by *B. thetaiotaomicron* has been investigated in monoculture, co-culture, and gnotobiotic mice^7^, and our previous study showed that YM increases the abundance of *B. thetaiotaomicron* in in vitro rat faeces fermentation^32^. However, the effects of YM on human colonic microbiota are still unknown. Here, we investigated the effects of YM on human colonic microbiota using an in vitro human faeces fermentation system (referred to as the Kobe University Human Intestinal Microbiota Model, or KUHIMM), which maintains the diversity and richness of bacterial species in the original human faeces^33^. The KUHIMM reproduces the effects of prebiotics, in line with the results from human clinical trials^34^. The use of the KUHIMM prior to human clinical trials allows us to evaluate the effects of YM on human colonic microbiota and the relevant doses, without the influence of dietary intake. In addition, we can also evaluate the safety of YM for unexpected microbiota changes, such as an increase in harmful bacteria, and the effects on the metabolic profile of each individual. Thus, the KUHIMM serves as a tool for evaluating the effect of functional food components, such as prebiotics, on human colonic microbiota.

## Results

### YM was utilised in the KUHIMM

YM was prepared from yeast cell wall slurry as described previously^32^, with a final mannan concentration of 50.5%. The KUHIMM was set up by adding a 0.4% YM preparation (0.2% mannan) (referred to as YM) under anaerobic conditions, and each of the eight human faecal samples (HS1, HS2, HS3, HS4, HS5, HS6, HS7, and HS8) (referred to as FEC) was used as the inoculum. A control culture without YM was also prepared (referred to as CUL). We investigated whether mannan was consumed by the human colonic microbiota in the KUHIMM after 30 h of fermentation. Mannan consumption was confirmed in all samples (Supplementary Fig. S1).

### YM administration did not alter bacterial genus-level composition and selectively stimulated the growth of *Bacteroides thetaiotaomicron* and *Bacteroides ovatus*

The effects of YM on human colonic microbiota were investigated using next-generation sequencing (NGS) and quantitative real-time PCR (qPCR). DNA was extracted from KUHIMM samples with and without YM collected after 30 h of fermentation. The eubacterial copy numbers, evaluated by qPCR, reached 2.81–4.90 × 10^11^ copies/mL (Supplementary Table S1), which were comparable to the reported cell densities in the human colon (approximately 10^11^ cells/mL)^35^.

NGS was used for the V3–V4 region of bacterial 16S rRNA for gene sequence analysis of faecal samples and the corresponding cultures with and without YM using the Illumina MiSeq system. In total, 4,407,318 quality reads were obtained from the eight faecal samples and the corresponding KUHIMMs with and without YM (Table 1). The numbers of operational taxonomic units (OTUs) and the Chao1 values for species richness were lower in the CUL and YM groups than in the FEC group (Kruskal–Wallis test, *p* < 0.05); however, there was no significant difference between the CUL and YM groups (Kruskal–Wallis test, *p* > 0.05). The Shannon index for species diversity was lower in the CUL group than in the FEC group (Kruskal–Wallis test, *p* < 0.05); however, there was no significant difference between the CUL and YM groups (Kruskal–Wallis test, *p* > 0.05). The Simpson index for species diversity was not significantly different among the FEC, CUL, and YM groups (Kruskal–Wallis test, *p* > 0.05). Thus, the microbial diversity in the KUHIMMs did not change with the addition of the 0.4% YM preparation.

**Table 1:** Summary of 16S rRNA gene sequencing data and α-diversity values (Chao1 estimator, Shannon index, and Simpson index).

	Faeces	KUHIMM	
		CUL	YM
Read counts	187,964 ± 19,718	183,017 ± 9224	179,934 ± 12,929
Observed OTUs	105.4 ± 16.1	78.6 ± 9.8*	76.1 ± 7.6*
Chao1	110.9 ± 18.4	85.0 ± 11.4*	81.6 ± 11.2*
Shannon index	4.24 ± 0.34	3.70 ± 0.19*	3.88 ± 0.23
Simpson index	0.90 ± 0.03	0.87 ± 0.04	0.89 ± 0.02

Eight human faecal samples, the corresponding culture without yeast mannan (CUL), and the corresponding culture with the 0.4% yeast mannan preparation (YM) were sampled 30 h after the initiation of fermentation. Values are the mean ± standard deviation. Asterisks (*) represent significant differences (*p < 0.05) (n = 8) between microbiota in the original faeces and the microbiota in corresponding cultures without or with yeast mannan using the Kruskal–Wallis test.

**Figure 2:**
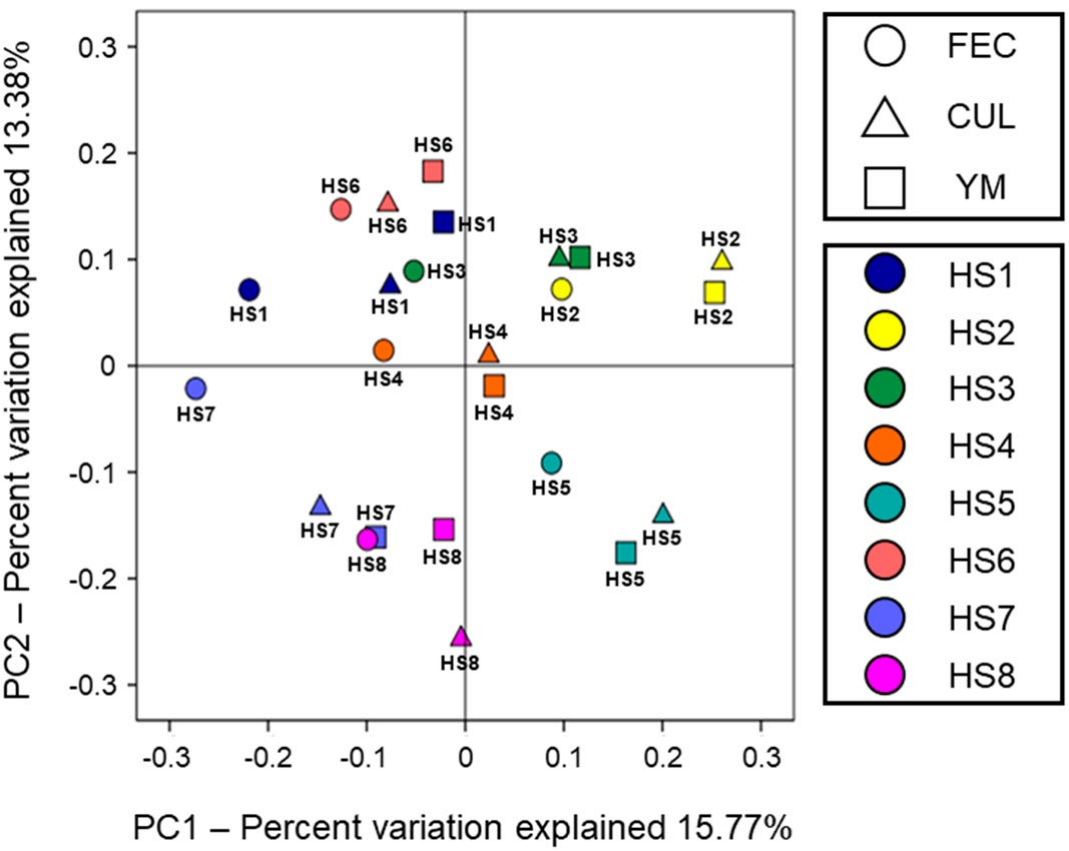
Principal coordinate analysis (PCoA) of 16S rRNA metagenomics data of bacterial species in eight human samples (HS1–HS8). Eight human faecal samples (FEC), the corresponding cultures without yeast mannan (CUL), and the corresponding cultures with a 0.4% yeast mannan preparation (YM) were sampled 30 h after the initiation of fermentation. The circles, triangles, and squares in the PCoA plot represent microbiota in the FEC, CUL, and YM groups, respectively.

**Figure 3:**
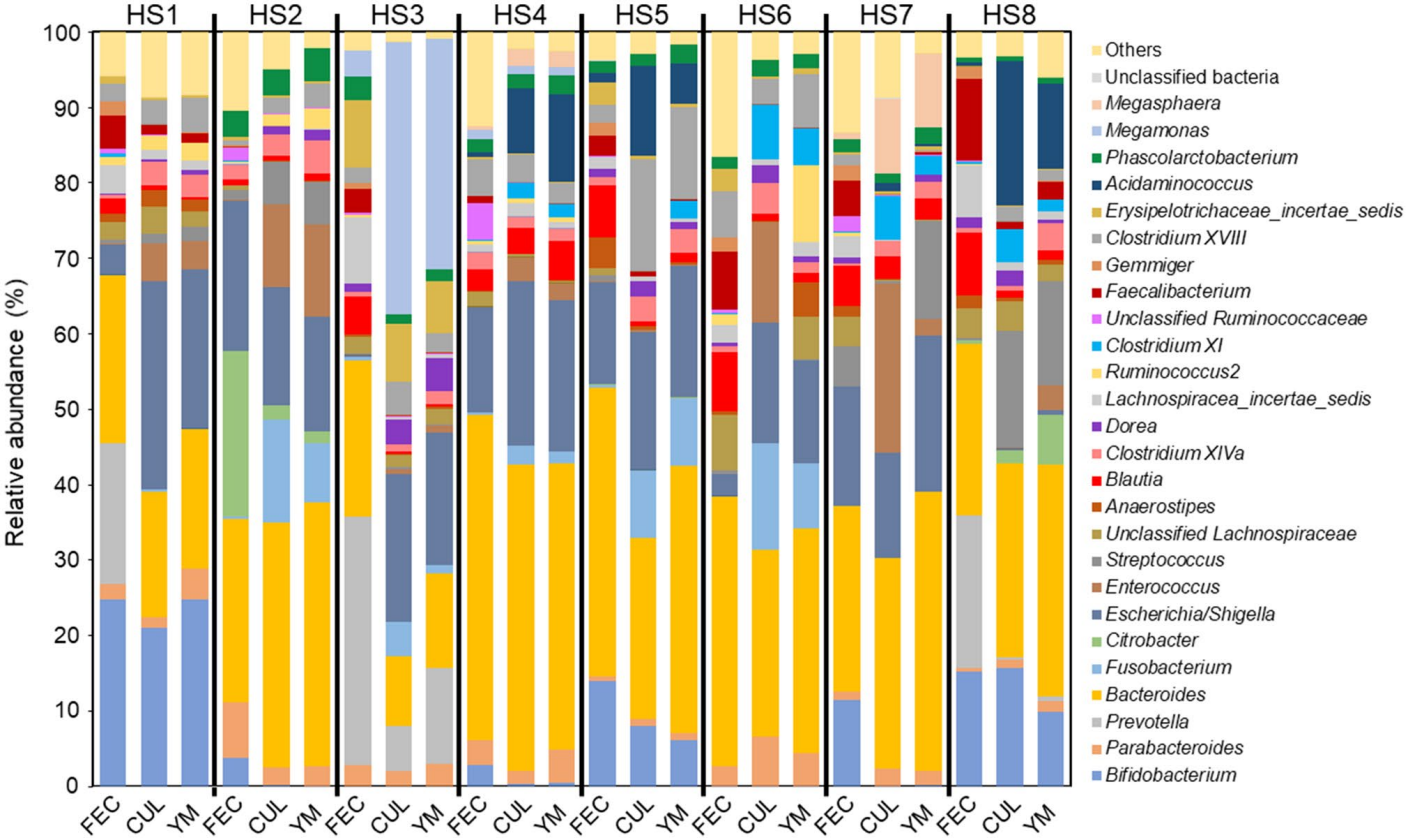
Genus-level compositional views of bacteria in eight human samples (HS1–HS8). Eight human faecal samples (FEC), the corresponding cultures without yeast mannan (CUL), and the corresponding cultures with the 0.4% yeast mannan preparation (YM) were sampled 30 h after the initiation of fermentation. Genera with low abundance (< 1.0%) and low similarity (< 97%) were included in ‘Others’ and ‘Unclassified bacteria’, respectively.

Principal coordinate analysis (PCoA) revealed that the microbiota in each KUHIMM was shifted in the same direction from the original faeces, and individual faecal samples and corresponding KUHIMMs with and without YM were assigned to the same cluster (Fig. 2). Microbiota β-diversity based on unweighted UniFrac distances was not significantly different between CUL and YM (permutational multivariate analysis of variance, PERMANOVA, *p* = 0.98). Bacterial genus-level compositional analyses of microbiota in the FEC, CUL, and YM are shown in Fig. 3. Almost all bacterial genera in the original faeces were also detected in the KUHIMMs. Comparing the relative abundance of 26 representative bacterial genera between CUL and YM, no significant differences were observed in any genus (Kruskal–Wallis test, *p* > 0.05). Thus, the microbial composition in the KUHIMMs did not change with the addition of the 0.4% YM preparation.

**Figure 4:**
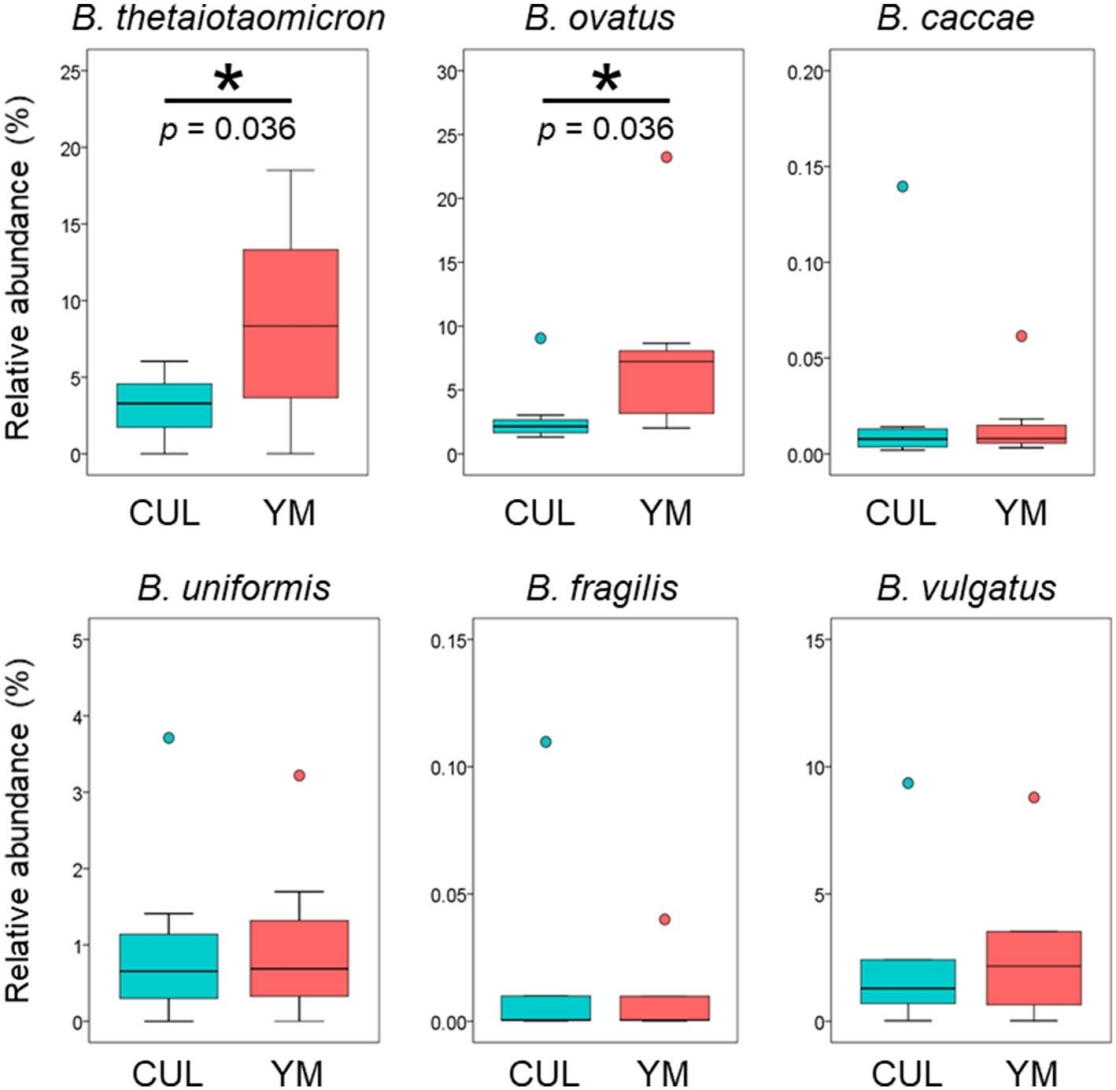
The relative abundance of six *Bacteroides* species after 30 h of fermentation in the KUHIMM. *Bacteroides thetaiotaomicron*, *Bacteroides ovatus*, *Bacteroides caccae*, *Bacteroides uniformis*, *Bacteroides fragilis*, and *Bacteroides vulgatus* without yeast mannan (CUL) and with the 0.4% yeast mannan preparation (YM) are shown. Asterisks (*) represent significant differences (*p < 0.05) (n = 7 for *B. uniformis*, n = 5 for *B. fragilis*, and *B. vulgatus*, n = 8 for others) using the Wilcoxon signed-rank test.

We then evaluated the effect of YM administration on bacteria of the genus *Bacteroides*, which are the most predominant anaerobes in the gut^36^, using the KUHIMM. *B. thetaiotaomicron*, *B. ovatus*, *B. caccae*, *B. uniformis*, *B. fragilis*, and *B. vulgatus* of the genus *Bacteroides* are commonly found at high densities in human colonic microbiota^37^. After 30 h of fermentation, the numbers of six *Bacteroides* species were estimated by qPCR analysis (Fig. 4). As expected, the relative abundance of *B. thetaiotaomicron* was significantly increased in the YM group compared to that in the CUL group (Wilcoxon signed-rank test, *p* = 0.036). Remarkably, the relative abundance of *B. ovatus* was also significantly increased in the YM group compared to that in the CUL group (Wilcoxon signed-rank test, *p* = 0.036). Conversely, the relative abundance of the other *Bacteroides* species, *B. caccae*, *B. uniformis*, *B. fragilis*, and *B. vulgatus*, was not significantly different (Wilcoxon signed-rank test, *p* = 0.48, 0.61, 0.69, and 0.35, respectively) between CUL and YM. Thus, *B. thetaiotaomicron* and *B. ovatus* in the KUHIMMs were selectively increased by the addition of the 0.4% YM preparation.

### YM administration reduced the pH and enhanced acetate and propionate production

**Figure 5:**
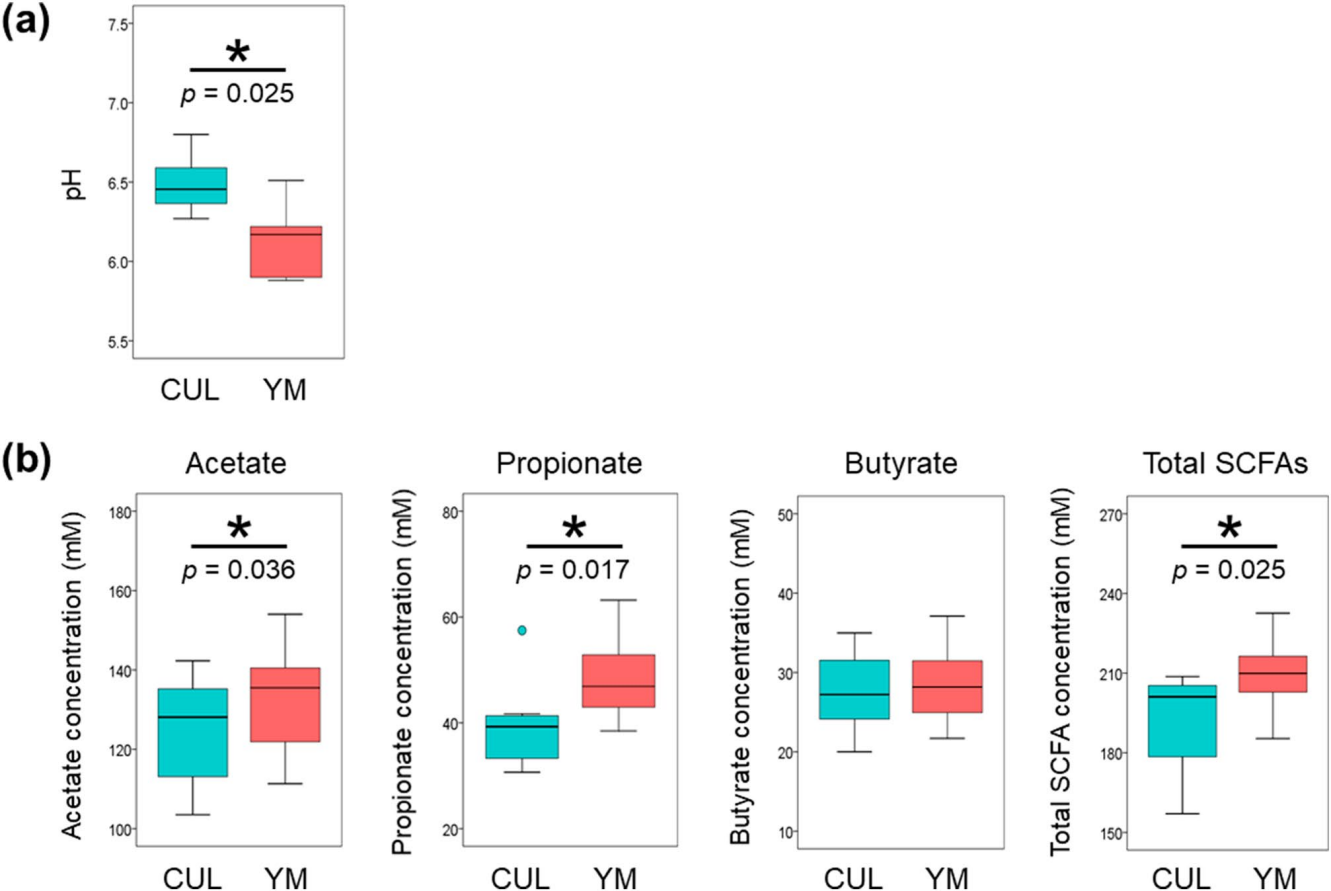
Changes in pH (**a**) and production of short-chain fatty acids (SCFAs) (**b**): acetate, propionate, butyrate, and total SCFAs after 30 h of fermentation in the KUHIMM without yeast mannan (CUL) and with the 0.4% yeast mannan preparation (YM). Asterisks (*) represent significant differences (*p < 0.05) (n = 8) using the Wilcoxon signed-rank test.

The pH reflects the intestinal environmental condition, and low pH inhibits the growth of pathogenic bacteria, resulting in the reduction of putrefactive compounds^38^. Supplementary Figure S2 shows the results of continuous monitoring of pH during culture. After 30 h of fermentation, the pH was significantly reduced in the presence of YM compared to that of the CUL group (Wilcoxon signed-rank test, *p* = 0.025, Fig. 5a).

SCFAs are metabolic products of human gut microbiota, which act as signalling molecules and provide beneficial effects for host health^39^. Acetate, propionate, and butyrate are the most abundant (≥ 95%) SCFAs in the human colon^40^. The impact of YM administration on the production of SCFAs was examined after 30 h of fermentation (Fig. 5b). The concentrations of acetate, propionate, and total SCFAs were significantly higher in the YM group than in the CUL group (Wilcoxon signed-rank test, *p* = 0.036, 0.017, and 0.025, respectively). In contrast, the concentration of butyrate was not significantly different between YM and CUL (Wilcoxon signed-rank test, *p* = 0.67).

## Discussion

The most recent definition of prebiotics is ‘a substrate that is selectively utilised by host microorganisms, conferring a health benefit’^41^, and numerous studies on prebiotics have found health benefits not only for the gut but also for the host in general^31,42^. Most traditional prebiotics increase the number of specific bacteria, such as *Lactobacillus* and *Bifidobacterium*^31^. In addition, they selectively increase the abundance at the bacterial genus level; few studies have reported on prebiotics that selectively increase abundance at the bacterial species level. It has been reported that bacteria of the genus *Bacteroides* have various beneficial effects^36^; among these bacteria, *B. thetaiotaomicron* and *B. ovatus* are expected to be utilised as potential novel probiotics^43,44^. Conversely, several species are pathogens and associated with harmful effects on host health, e.g. *B. fragilis* with the induction of abscess formation^45^ and *B. vulgatus* with the development of ulcerative colitis^46^. Therefore, a product that selectively increases beneficial bacteria of the genus *Bacteroides* could be a functional food ingredient as a novel prebiotic candidate.

In this study, we investigated the effect of YM on human colonic microbiota and metabolic end products using an in vitro human faeces fermentation system, the KUHIMM. Bacterial 16S rRNA gene sequence analysis showed that YM administration did not change microbial α-diversity, β-diversity, or the relative abundance of representative bacterial genera. Analysis of the growth profiles of six *Bacteroides* species in the KUHIMM revealed that YM administration stimulated the growth of only two species, *B. thetaiotaomicron* and *B. ovatus*, through the consumption of mannan. These results indicate that YM selectively increases the abundance of *B. thetaiotaomicron* and *B. ovatus*. To the best of our knowledge, there are few prebiotics that increase microbes in a species-specific manner, and YM is the first material that selectively increases *B. thetaiotaomicron* and *B. ovatus* in the complex of human colonic microbiota. *B. ovatus* is reported to exhibit immunogenic and immunomodulatory functions, such as expression of the tumour-specific Thomsen–Friedenreich antigen as a target for a cancer vaccine^47^ and alleviation of lipopolysaccharide-induced inflammation^48^. In addition to *B. thetaiotaomicron*, several strains of *B. ovatus*, *B. vulgatus*, and *B. caccae* metabolised mannan in monoculture^7^, although among these species, only *B. ovatus* was increased in human colonic microbiota.

**Figure 6:**
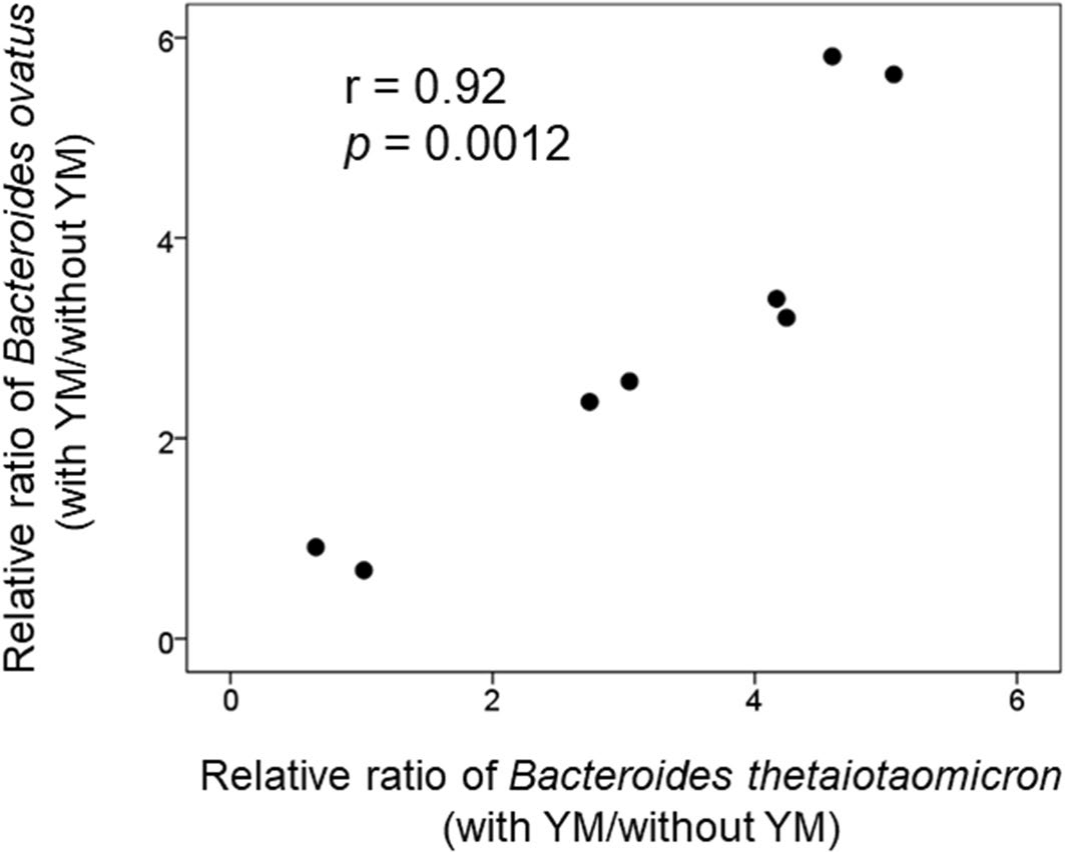
The correlation between the relative ratio of *Bacteroides thetaiotaomicron* and *Bacteroides ovatus* (with the 0.4% yeast mannan preparation [YM]/without yeast mannan [CUL]) in the KUHIMM after 30 h of fermentation (n = 8).

Both *B. thetaiotaomicron* and *B. ovatus* can degrade various indigestible polysaccharides utilising Sus-like systems^22^. These bacteria break down polysaccharides extracellularly to liberate polysaccharide breakdown products (PBPs). Some of them produce PBPs exclusively for their own use, while others produce PBPs that they do not necessarily require but can be used for growth by other *Bacteroides* spp. having limited or no ability to use the polysaccharides^23,49,50^. In addition, there are potential effects outside the genus *Bacteroides*; *B. ovatus* liberates PBPs during growth on xylan, which can support the growth of *Bifidobacterium adolescentis* that are normally unable to utilise it^51^. One study showed that *B. thetaiotaomicron* uses YM exclusively through a selfish mechanism, in which *B. thetaiotaomicron* degrades YM into PBPs extracellularly and incorporates them into the cell in a manner that prevents other bacteria from using it, implying that *B. thetaiotaomicron* does not support the growth of other *Bacteroides* that can use the mannose- and mannan backbone^7^. In addition, *B*. *thetaiotaomicron* grew more efficiently than *B. ovatus* on YM in monoculture^22^. However, unlike what was previously thought, we found the interesting result that YM increased both *B. thetaiotaomicron* and *B. ovatus* to the same extent. Notably, the relative abundances of *B. thetaiotaomicron* and *B. ovatus* in the YM group relative to the control group had a strong positive correlation (r = 0.92, *p* = 0.0012, Fig. 6), although there were no significant correlations between the relative abundances of *B. thetaiotaomicron* and *B. ovatus* in the culture (r =  − 0.05, *p* = 0.86). Therefore, it is considered that *B*. *thetaiotaomicron* and *B. ovatus* utilise YM in a coordinated manner, rather than in a competitive manner.

**Figure 7:**
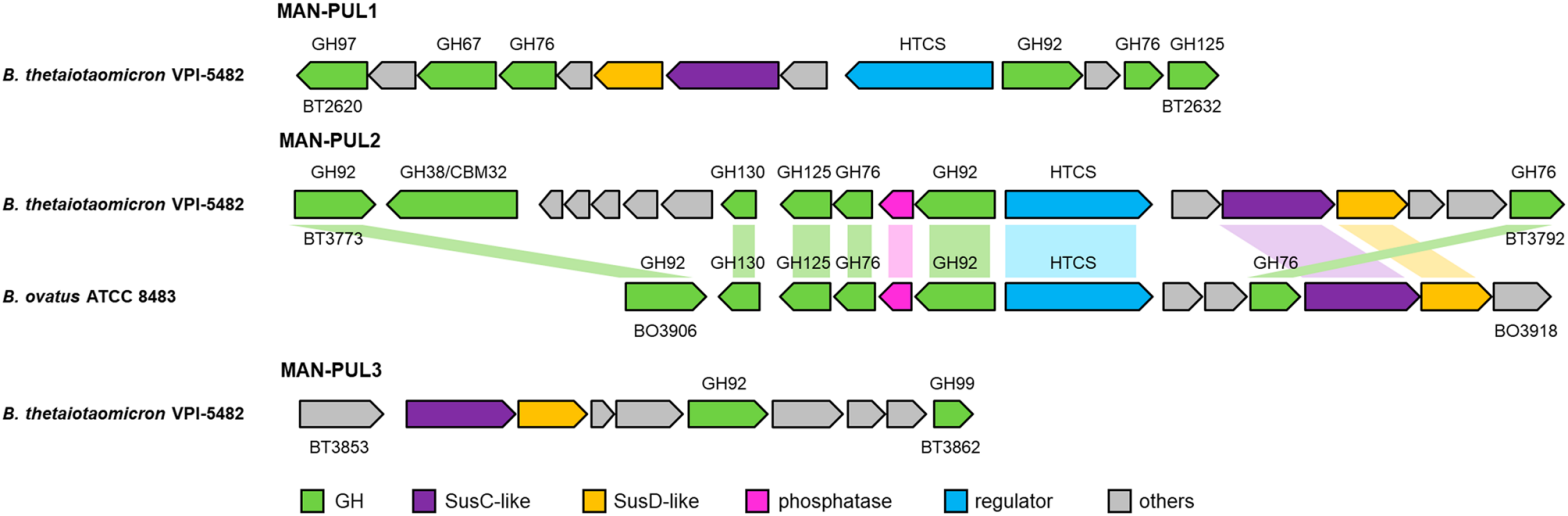
*Bacteroides thetaiotaomicron* VPI-5842 and *Bacteroides ovatus* ATCC 8483 yeast mannan polysaccharide utilisation loci (PULs). Each gene is depicted to reflect its orientation and scale on the genome. The number below each gene is its locus tag. Genes encoding known or predicted functionalities are colour-coded: glycoside hydrolase (GH; green), SusC-like proteins (SusC-like; purple), SusD-like proteins (SusD-like; orange), phosphatase (phosphatase; pink), regulatory proteins (regulator; blue), and other proteins (others; grey).

Three putative PULs (MAN-PUL1, MAN-PUL2, and MAN-PUL3) are important for *B. thetaiotaomicron* to utilise YM as the sole carbon source^7^. Bioinformatics studies found that *B. ovatus* possesses a putative PUL corresponding to MAN-PUL2 but no PULs corresponding to MAN-PUL1 or MAN-PUL3 in its genome (Fig. 7, Supplementary Table S2). A model has been proposed wherein YM is degraded extracellularly by at least two GH76s (endo-α-1, 6-mannanases BT2623 and BT3792) and a GH99 (endo-α-1, 2-mannosidase and endo-α-1, 2-mannanase BT3862) within these PULs to liberate PBPs, which are then transported into the periplasm, where they are depolymerised to mannose^7^. Of these three proteins, *B. ovatus* possesses only one GH76 (BO3915), with a relatively lower degree of homology with the other GHs in MAN-PUL2, suggesting that the extracellular degradation of YM is incomplete. Thus, in the in vitro human colonic microbiota model, *B*. *ovatus* appears to have utilised PBPs generated by *B. thetaiotaomicron* from the YM by incorporating them into the periplasm, revealing a novel cooperative relationship between *Bacteroides* species. This interesting phenomenon might have evolved cooperatively between *B. thetaiotaomicron* and *B. ovatus* in complex gut microbial ecosystems where various microbes compete for limited nutrients.

An increase in the production of acetate, propionate, and total SCFAs was observed in the culture with YM. The phylum Bacteroidetes is known to primarily produce acetate and propionate as metabolic end products^40^. Therefore, it was suggested that YM administration stimulated the growth of *B. thetaiotaomicron* and *B. ovatus*, increased the relative abundance of the phylum Bacteroidetes, and resulted in an increase in acetate and propionate. These SCFAs are thought to have reduced the pH. Acetate and propionate are the most potent activators of GPR43, a receptor on the cell surfaces of adipose tissue^52^. Because one SCFA is utilised by intestinal bacteria to produce another SCFA, and changes in the intestinal microbiota compositions are associated with the production of SCFAs^39^, an increase in one SCFA ideally should not reduce the levels of another beneficial SCFA. Therefore, YM might be a useful prebiotic because it increased the production of acetate, propionate, and total SCFAs and did not decrease that of butyrate.

Because there are various factors in the complex intestinal microbial ecosystem, including competition for prebiotics and cross-feeding interactions among microorganisms, even if a material is utilised by certain intestinal bacteria in monoculture, it may not necessarily increase the bacteria in the intestinal microbiota. Furthermore, the intake of prebiotics by humans may cause a considerable increase in the abundance of bifidobacteria, even if they do not affect the growth of bifidobacteria in in vitro monoculture^53^. Therefore, it was important to confirm that YM selectively increased *B. thetaiotaomicron* and *B. ovatus* abundance in the in vitro human colonic microbiota fermentation system, which reproduces the in vivo microbiota changes induced by prebiotics. The prebiotic effects of YM were confirmed at doses as low as 0.4% (0.2% mannan). Previous studies using this system have confirmed the bifidogenic effects of prebiotic oligosaccharides at a concentration of 0.5%^34^, while 0.2% did not change the colonic microbiota composition as reported in human and animal studies^33^. For this reason, compared to conventional prebiotics, YM may also exert prebiotic effects at lower doses in in vivo human clinical studies. However, when YM is ingested by humans, it may be affected by variation in diets; therefore, it is not clear whether YM exhibits the same prebiotic effect. To develop YM as a microbiota-directed food ingredient for human consumption that selectively increases the abundance of *B. thetaiotaomicron* and *B. ovatus*, clinical studies are required to verify its prebiotic effect, the resulting health benefits, and the doses at which these effects are produced.

## Conclusion

YM selectively increased the relative abundance of *B. thetaiotaomicron* and *B. ovatus* in the human colonic microbiota model. In addition, YM increased the production of acetate, propionate, and total SCFAs. These results show the potential of YM as a novel prebiotic that selectively increases *B. thetaiotaomicron* and *B. ovatus* and improves the intestinal environment.

## Methods

### Preparation of YM

YM was produced from yeast cell wall slurry provided by Asahi Group Foods, Ltd. (Tokyo, Japan) as described previously^32^. The mannan concentration was measured by Japan Food Research Laboratories (Tokyo, Japan). The mannan concentration was 50.5%, calculated based on the mannose concentration after hydrolysis, which was quantified by high-performance liquid chromatography (HPLC).

### Human faecal sample collection from volunteers

Faecal samples were obtained from eight healthy subjects in their thirties to forties who had not taken antibiotics for at least 2 months prior to sampling, as described previously^33^. Written informed consent was obtained from all participants. The study was performed in accordance with the principles of the Declaration of Helsinki and the guidelines of Kobe University and was approved by the Intestinal Ethics Review Board at Kobe University (research code 1902, approval date 10 May 2016). All methods used in this study were in accordance with the guidelines of the Medical Ethics Committee at Kobe University. Approximately 200 mg faecal samples were collected using anaerobic culture swabs (212559 BD BBL CultureSwab; BD Biosciences, Franklin Lakes, NJ, USA) and stored at room temperature according to the manufacturer’s protocol until inoculation.

### Operation of the KUHIMM with and without YM

The model culture system was operated using a multi-channel fermenter (Bio Jr. 8; ABLE, Tokyo, Japan) to construct the KUHIMM as described previously^33,34^. Briefly, each vessel in the system contained autoclaved Gifu anaerobic medium broth (100 mL; Nissui Pharmaceutical Co.), with the initial pH adjusted to 6.5. Anoxic conditions were established by purging vessels with a filter-sterilised mixture of N_2_ and CO_2_ (80:20) gas (15 mL/min) at 37 °C prior to and during fermentation. To prepare the inoculum, each faecal sample was suspended in 2 mL of 0.1 M phosphate buffer (pH 6.5, 0.1 M NaH_2_PO_4_:0.1 M Na_2_HPO_4_ = 2:1), supplemented with 1% l-ascorbic acid (Wako Pure Chemical Industries, Osaka, Japan). Fermentation was initiated by inoculation of each medium-containing vessel with 100 μL of the abovementioned faecal suspension. To evaluate the effects of YM administration, YM was added into one of the vessels at a final concentration of 4 g/L (0.4% per 100 mL of medium) prior to fermentation. A control vessel without YM was also prepared. Faecal samples and aliquots of fermentation cultures were stored at − 20 °C until use.

### Extraction of microbial genomic DNA

Microbial DNA was extracted from faecal samples and fermentation cultures of the KUHIMM using 0.1 mm glass beads, TE (10 mM Tris–HCl and 1 mM ethylenediaminetetraacetic acid [EDTA])-saturated phenol, and sodium dodecyl sulphate, as described previously^34^. A OneStep PCR Inhibitor Removal Kit (Zymo Research, Irvine, CA, USA) was used for further purification according to the manufacturer’s instructions. Purified DNA was stored at − 20 °C until use.

### Next-generation sequencing and data processing

NGS analysis was performed by Macrogen Japan Corp. (Kyoto, Japan). Samples for sequencing were prepared according to the Illumina 16S Metagenomic Sequencing Library Preparation protocols to amplify the V3 and V4 regions of the 16S rRNA genes. Bacterial 16S rRNA genes (V3–V4 region) were amplified using genomic DNA as the template. The following primers were used: S-D-Bact-0341-b-S-17 (5′-CCTACGGGNGGCWGCAG-3′) and S-D-Bact-0785-a-A-21 (5′-GACTACHVGGGTATCTAATCC-3′)^54^. PCR was performed according to the manufacturer’s instructions. Amplicons were purified using AMPure XP beads (Beckman Coulter, Inc., CA, USA). Paired-end sequencing was performed on the Illumina MiSeq platform. Overlapping reads were merged using fast length adjustment of short reads^55^. Pre-processing and clustering of sequences to identify OTUs was performed using the CD-HIT-OTU software^56^. After short reads were filtered out and extra-long tails were trimmed, chimeric reads were identified and discarded. The remaining representative reads were clustered into OTUs based on a ≥ 97% similarity threshold. Taxonomic composition for each sample from phylum to species levels was generated using QIIME-UCLUST^57^ against the RDP-16S rRNA gene database^58^. The various α-diversity values (Chao1, Shannon index, and Simpson index) and PCoA of unweighted UniFrac distances^59^ were calculated using QIIME software^60^.

### Quantitative real-time PCR

Quantitative real-time PCR (qPCR) analyses were performed using specific primers for all eubacteria and six *Bacteroides* species (*B. thetaiotaomicron*, *B. ovatus*, *B. caccae*, *B. uniformis*, *B. fragilis*, and *B. vulgatus*) as described previously^37,61^. qPCR analyses were conducted in duplicate using an Applied Biosystems QuantStudio 3 Real-Time PCR system (Thermo Fisher Scientific Inc., Waltham, MA, USA). The qPCR amplification program is described in Supplementary Table S3. We prepared a synthesised DNA fragment (191–265 bp) identical to the 16S rRNA gene sequence as a reference for absolute quantification for each method. Standard curves were prepared by diluting reference fragments (10^1^–10^8^ copies). To confirm the specificity of the amplification using SYBR Green, a melting-point-determination analysis was performed.

### Measurement of mannan concentration in the model culture system

The remaining mannan in the culture medium was analysed at 0 h and 30 h after the initiation of fermentation and was calculated based on the mannose concentration after hydrolysis, which allows the measurement of mannan utilisation. Samples were prepared according to the method described by Goubet et al.^62^ with minor modification. Briefly, each culture broth was centrifuged at 10,000 × *g* for 5 min, and 100 μL of the supernatant was recovered, which was then hydrolysed using 1 mL of 2 M trifluoroacetic acid for 4.5 h at 100 °C. The samples were combined with 1 mL of 99.5% ethanol and dried using a centrifugal evaporator (Genevac Ltd., Ipswich, UK). The dry residue was resuspended in water, and the low-molecular-weight (< 10 kDa) fraction was recovered using Vivaspin 500 MWCO 10,000 PES (Sartorius Stedim Biotech, Göttingen, Germany).

The mannose concentration was determined by high-performance anion-exchange chromatography with a pulsed amperometric detector (HPAEC-PAD). HPAEC-PAD analysis was performed using a Dionex ICS-5000 (Thermo Fisher Scientific, CA, USA). The system was equipped with a CarboPac PA1 column (2 × 250 mm) in combination with a CarboPac PA1 guard column (2 × 50 mm) (Thermo Fisher Scientific). The mobile phases consisted of 10 mM NaOH (A) and 500 mM NaOH (B). Samples (10 μL) were applied to the column and eluted at a flow rate of 0.25 mL/min using the following linear gradient: 0 min–0% B; 20 min–0% B; 20.01 min–60% B; 35 min–60% B; 35.01 min–0% B; 50 min–0% B.

### Measurement of SCFA concentrations

The concentrations of lactate, succinate, acetate, propionate, and butyrate were measured using an HPLC system (Shimadzu, Kyoto, Japan) equipped with an Aminex HPX-87H column (Bio-Rad Laboratories, Hercules, CA, USA) and an RID-10A refractive index detector (Shimadzu), as described previously^33^.

### Bioinformatics and statistical analyses

PULs similar to MAN-PUL1, MAN-PUL2, and MAN-PUL3 were searched using the PUL prediction tool described in PULDB^63^. The Kruskal–Wallis test and Wilcoxon signed-rank test were performed using SPSS software ver. 23 (IBM Japan, Ltd., Tokyo, Japan). PERMANOVA was performed using the R ver. 3.6.0 Vegan package. A *p*-value < 0.05 was considered statistically significant.

## Supplementary information

Supplementary Information.
