# Author Correction: Prebiotic effects of yeast mannan, which selectively promotes *Bacteroides thetaiotaomicron* and *Bacteroides ovatus* in a human colonic microbiota model

**DOI:** 10.1038/s41598-021-83010-9

**Published:** 2021-02-08

**Authors:** Shunsuke Oba, Tadahiro Sunagawa, Reiko Tanihiro, Kyoko Awashima, Hiroshi Sugiyama, Tetsuji Odani, Yasunori Nakamura, Akihiko Kondo, Daisuke Sasaki, Kengo Sasaki

**Affiliations:** 1 Core Technology Laboratories, Asahi Quality & Innovations, Ltd, 1-21, Midori 1-Chome, Moriya-Shi, 302-0106 Japan; 2 Graduate School of Science, Technology and Innovation, Kobe University, 1-1 Rokkodai-cho, Nada-ku, Kobe, Hyogo 657-8501 Japan

Correction to: *Scientific Reports* https://doi.org/10.1038/s41598-020-74379-0, published online 15 October 2020

This Article contains an error in Figure 1 where the colours for mannose and *N*-acetyl-D-glucosamine (GlcNAc) are incorrect.

The correct Figure 1 appears below.

**Figure 1:**
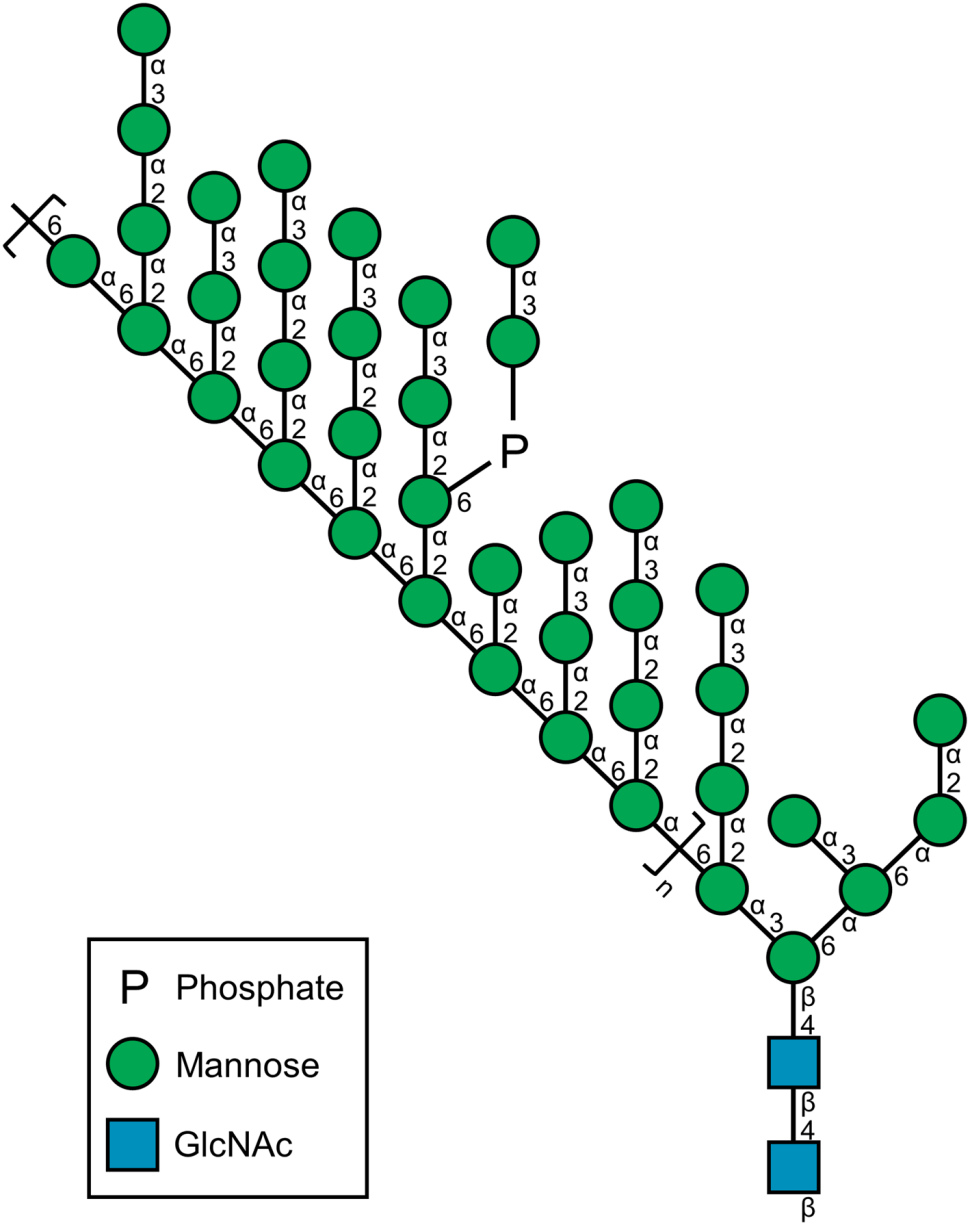
A correct version of the original Figure 1.

