# Supplementary material for: Prebiotic effects of yeast mannan, which selectively promotes Bacteroides thetaiotaomicron and Bacteroides ovatus in a human colonic microbiota model
Source: Sci Rep. 2020 Oct 15;10:17351. doi: 10.1038/s41598-020-74379-0 (PMC7562712; doi:10.1038/s41598-020-74379-0)
Supplement: Supplementary file 1 — Supplementary Information. [file 41598_2020_74379_MOESM1_ESM.pdf]

Supplementary Information

Title

Prebiotic effects of yeast mannan, which selectively promotes *Bacteroides thetaiotaomicron* and *Bacteroides ovatus* in a human colonic microbiota model

Shunsuke Oba[1]*, Tadahiro Sunagawa[1], Reiko Tanihiro[1], Kyoko Awashima[1], Hiroshi Sugiyama[1], Tetsuji Odani[1], Yasunori Nakamura[1], Akihiko Kondo[2], Daisuke Sasaki[2], & Kengo Sasaki[2]

[1]Core Technology Laboratories, Asahi Quality & Innovations, Ltd., Ibaraki 302-0106, Japan

[2]Graduate School of Science, Technology and Innovation, Kobe University, 1-1 Rokkodai-cho, Nada-ku, Kobe, Hyogo 657-8501, Japan

*Corresponding author: Shunsuke Oba; Core Technology Laboratories, Asahi Quality & Innovations, Ltd., 1-21, Midori 1-Chome, Moriya-Shi 302-0106, Japan; Tel: +81-297-46-9383; Email: shunsuke.oba@asahi-qi.co.jp

Supplementary Table S1.

Eubacterial copy numbers in the Kobe University Human Intestinal Microbiota Model (KUHIMM) after 30 h of fermentation without yeast mannan (CUL) and with the 0.4% yeast mannan preparation (YM).

	CUL ($\times 10^{11}$ copies/mL)	YM ($\times 10^{11}$ copies/mL)
HS1	3.91	3.75
HS2	4.90	4.24
HS3	3.94	4.43
HS4	4.25	4.74
HS5	4.11	4.70
HS6	3.20	4.21
HS7	2.83	4.36
HS8	2.81	4.87

Supplementary Table S2

Sequence identity and similarity of proteins encoded on MAN-PUL2 for two strains, *Bacteroides thetaiotaomicron* VPI-5842 and *Bacteroides ovatus* ATCC 8483.

B. thetaiotaomicron VPI-5482 MAN-PUL2 proteins	*B. ovatus* ATCC 8483 MAN-PUL2 proteins	Sequence identity (%)	Sequence similarity (%)
BT3773 (GH92)	BO3906 (GH92)	91	94
BT3780 (GH130)	BO3907 (GH130)	87	95
BT3781 (GH125)	BO3908 (GH125)	92	95
BT3782 (GH76)	BO3909 (GH76)	86	91
BT3783 (phosphatase)	BO3910 (phosphatase)	88	93
BT3784 (GH92)	BO3911 (GH92)	91	96
BT3786 (HTCS)	BO3912 (HTCS)	82	91
BT3792 (GH76)	BO3915 (GH76)	31	43
BT3788 (SusC-like)	BO3916 (SusC-like)	34	52
BT3789 (SusD-like)	BO3917 (SusD-like)	25	39

Supplementary Table S3

qPCR amplification conditions for each bacterial species.

Target species	Primer-F<br>Primer-R (5' to 3')	Strains for<br>standard curves	PCR amplification condition	Ref.
all eubacteria	CGGTGAATACGTTCCCGG<br>TACGGCTACCTTGTTACGACTT	*B. fragilis* ATCC 25285	95 °C for 10 sec,<br>[95 °C for 20 sec, 56 °C for 20 sec, and 72 °C for 30 sec] × 40 cycles	61
B. thetaiotaomicron	GCAAACTGGAGATGGCGA<br>AAGGTTTGGTGAGCCGTTA	*B. thetaiotaomicron* ATCC 29741	95 °C for 30 sec,<br>[95 °C for 15 sec and 62.5 °C for 60 sec] × 40 cycles	37
B. ovatus	TGCAAACTRAAGATGGC<br>CAAACTAATGGAACGCATC	*B. ovatus* ATCC 8483	95 °C for 30 sec,<br>[95 °C for 15 sec and 58 °C for 60 sec] × 40 cycles	37
B. caccae	AAACCCATACGCCGCAAG<br>GACACCTCACGGCACGAG	*B. caccae* ATCC 43185	95 °C for 30 sec,<br>[95 °C for 15 sec and 63 °C for 60 sec] × 40 cycles	37
B. uniformis	TCTTCCGCATGGTAGAACTATTA<br>ACCGTGTCTCAGTTCCAATGTG	*B. uniformis* ATCC 8492	95 °C for 30 sec,<br>[95 °C for 15 sec and 60 °C for 60 sec] × 40 cycles	37
B. fragilis	AGGATTCCGGTAAAGGATGG<br>GTTCAGGCTAGCGCCCATT	*B. fragilis* ATCC 25285	95 °C for 30 sec,<br>[95 °C for 5 sec and 64 °C for 30 sec] × 35 cycles	This study
B. vulgatus	CGGGCTTAAATTGCAGATGA<br>CATGCAGCACCTTCACAGAT	*B. vulgatus* ATCC 8482	95 °C for 30 sec,<br>[95 °C for 15 sec and 63 °C for 60 sec] × 40 cycles	37

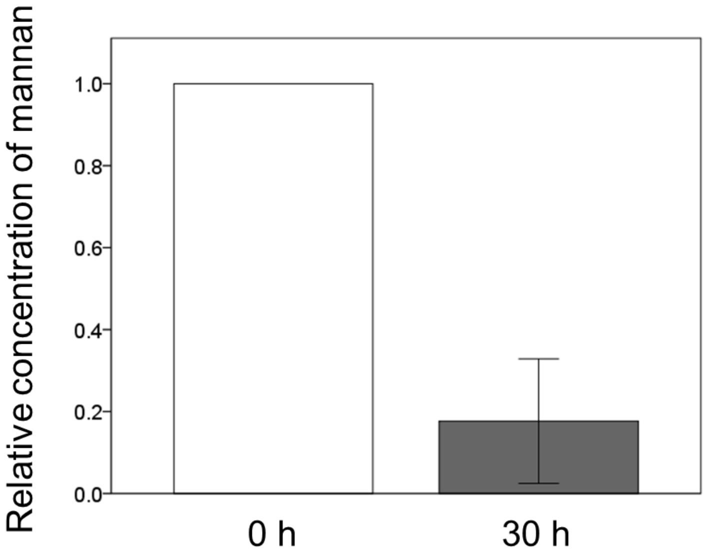

Supplementary Figure S1

Changes in the concentration of mannan between 0 h and 30 h after the initiation of fermentation with 0.2% mannan in the culture medium. Fermentation was initiated by inoculating each of the human faecal samples obtained from eight healthy subjects (HS1–HS8). Changes are presented as the ratio of the mannan concentration in the Kobe University Human Intestinal Microbiota Model (KUHIMM) at 30 h (grey) to those at 0 h (white). Error bars show the standard deviation of the mean.

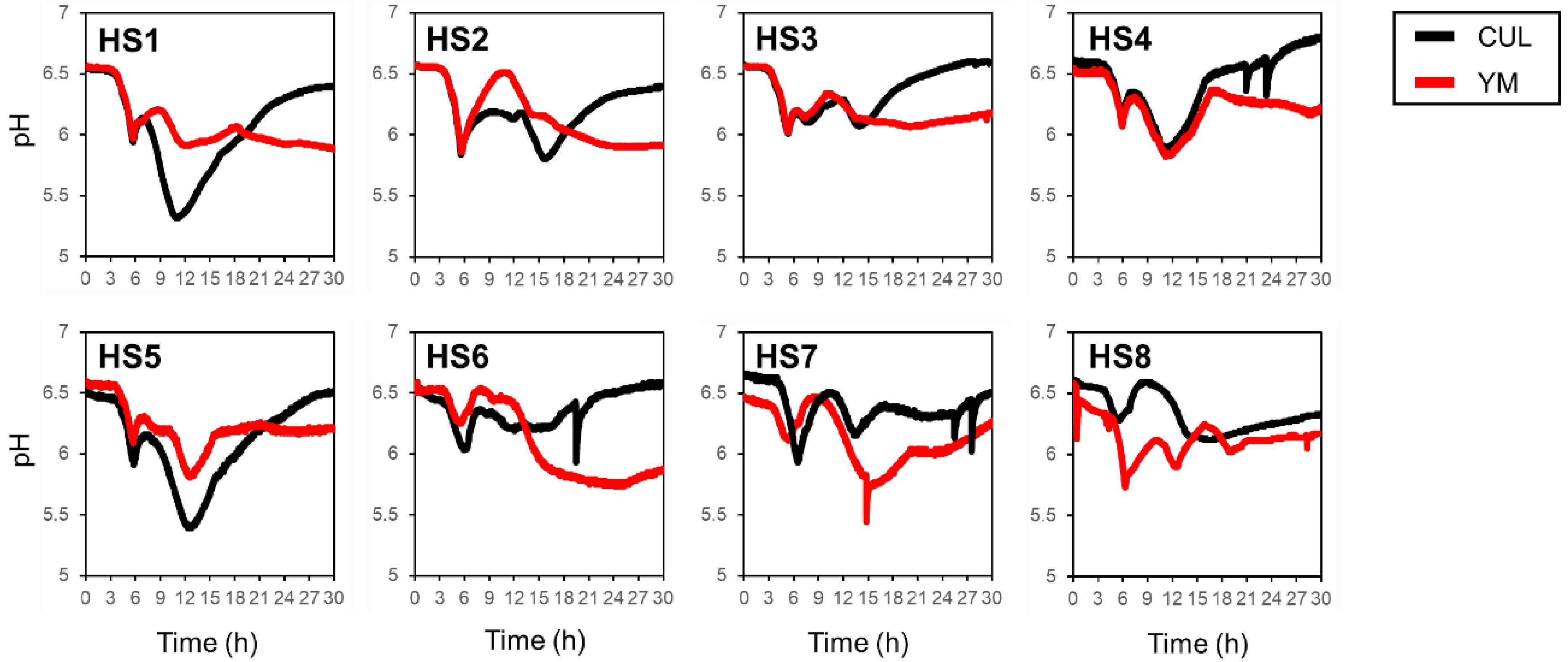

Supplementary Figure S2

Time-dependent pH profiles obtained during fermentation in the Kobe University Human Intestinal Microbiota Model (KUHIMM) without yeast mannan (CUL) and with the 0.4% yeast mannan preparation (YM). Fermentation was initiated by inoculating each of the human faecal samples obtained from eight healthy subjects (HS1–HS8).
